# Rapid Determination of Methamphetamine, Methylenedioxymethamphetamine, Methadone, Ketamine, Cocaine, and New Psychoactive Substances in Urine Samples Using Comprehensive Two-Dimensional Gas Chromatography

**DOI:** 10.3390/metabo14110643

**Published:** 2024-11-20

**Authors:** Doreen N. B. Chandra Siri, Seng Yo Goh, Ngee Sing Chong, Philip J. Marriott, Yong Foo Wong

**Affiliations:** 1 Center for Research on Multidimensional Separation Science, School of Chemical Sciences, Universiti Sains Malaysia, USM 11800, Penang, Malaysia; 2 Toxicology Section, Forensic Division, Department of Chemistry Malaysia, George Town 10450, Penang, Malaysia; 3 Agilent Technologies Sales (M) Sdn. Bhd., Unit 202 & 203, Level 2, Uptown 2, Jalan SS21/37, Damansara Uptown, Petaling Jaya 47400, Selangor, Malaysia; 4 Department of Chemistry, Middle Tennessee State University, Murfreesboro, TN 37132, USA; 5 Australian Centre for Research on Separation Science, School of Chemistry, Monash University, Wellington Road, Clayton Victoria 3800, Australia

**Keywords:** new psychoactive substances, comprehensive two-dimensional gas chromatography, methamphetamine, mephedrone, α-pyrrolidinovalerophenone, ecstasy, ephylone

## Abstract

**Background/Objectives:** This study evaluates the applicability of a comprehensive two-dimensional gas chromatography−flame ionisation detection (GC×GC−FID) approach for the simultaneous determination of 12 underivatised psychoactive drugs, including new psychoactive substances, that comprised of amphetamine, methamphetamine, mephedrone, 3,4-methylenedioxyamphetamine, 3,4-methylenedioxymethamphetamine, α-pyrrolidinovalerophenone, *n*-ethylpentylone (ephylone), norketamine, ketamine, 3,4-methylenedioxypyrovalerone, methadone, and cocaine. **Methods:** Separation was effected using a non-polar first dimension (^1^D) and a polar second dimension (^2^D) column, demonstrating an improved separation of drug compounds compared to a polar/non-polar column configuration. Interference-free baseline separation of all psychoactive compounds in a urine matrix was achieved within 8 min. The GC×GC−FID method was validated according to the guidelines defined by Standard Practices for Method Validation in Forensic Toxicology. **Results:** The calibration curves for the 12 psychoactive drugs were well correlated (r^2^ > 0.99) within the concentration ranges of 50–1500 ng mL^−1^. Detection limits of 10–20 ng mL^−1^ were obtained, and good repeatability and reproducibility (CV < 11.4%) were attained for retention times and peak areas. Method recoveries for the small-scale solvent extraction procedure ranged from 96.9 to 114.5%, and bias was between −3.1% and 14.5%. **Conclusions:** The validated approach was successfully applied for the determination of these illicit compounds in spiked urine samples of different concentrations, highlighting its potential for rapid forensic drug screening.

## 1. Introduction

The global trend in drug abuse indicates a persistent increase in stimulant dependence, with millions of individuals reportedly injecting opioids, amphetamines, methamphetamines, and cocaine, amongst other drugs, intravenously [1]. This widespread psychoactive substance abuse detrimentally impacts public health, societal stability, and safety and imposes significant economic ramifications [2,3,4]. The deleterious effects of drug abuse include dependence, transmission of disease such as human immunodeficiency virus, tuberculosis, and hepatitis C, mental health disorders, heightened violence and crime, diminished quality of life, and increased mortality [5,6,7,8]. Phenethylamines such as methamphetamine and 3,4-methylenedioxymethamphetamine (MDMA, also known as “ecstasy”) rank among the most widely abused psychoactive substances worldwide, following trends in cannabinoids and opioids [9]. While heroin continues to be the most prevalent opioid of abuse, the misuse of synthetic opioids has surged significantly. Synthetic opioids, such as methadone, possess potency many times greater than heroin and morphine and are increasingly linked to fatal drug overdoses [5,10]. Ketamine, which is used as a therapeutic drug to treat patients with depression, has been linked to overdoses and deaths [11]. Most recently, the United States Department of Justice has filed charges against two doctors involved in the distribution of ketamine in the ketamine overdose death investigation of an actor [12].

Recent trends in drug manufacturing indicate a significant shift with the emergence of designer drugs or new psychoactive substances (NPS), driven by their availability through international distribution and online markets [13]. The United Nations Office on Drugs and Crime (UNODC) Early Warning Advisory on New Psychoactive Substances (2023) reported that 1230 NPS have been identified hitherto, raising significant concern across 141 countries [14]. Most of these substances are stimulants designed to mimic the effects of controlled illicit substances such as cannabis, cocaine, and methamphetamine. Mephedrone, α-pyrrolidinovalerophenone (PVP), *n*-ethylpentylone (ephylone), and 3,4-methylenedioxypyrovalerone (MDPV) are examples of NPS comprising of synthetic cathinones, cannabinoids, phencyclidine-type substances, phenethylamines, piperazines, tryptamines, aminoindanes, plant-based substances, benzodiazepines, fentanyl analogues, and others [6,15]. The intrinsic complexity of biological specimens, with the increasing number of chemical compounds with novel structures in the drug market, makes the determination of these substances in biological samples highly challenging [16,17]. Given the lack of information regarding the potency, pharmacological effects, and toxicological safety of NPS, their misuse raises significant health concerns, amid apprehensions that these substances offer no medical benefits and could potentially be detrimental to health [18,19].

The complexity of bioanalysis highlights the challenges in detecting drugs and their metabolites, primarily attributed to the trace concentrations of xenobiotics and the intricate nature of the biological matrix [20,21,22]. Hence, there is a need for methodologies that provide reliable and accurate information, driving the continuous advancement of analytical techniques for the qualitative and quantitative determination of psychoactive substances in biological matrices. Chromatographic techniques, particularly gas chromatography (GC) and high-performance liquid chromatography (HPLC), coupled with various detection modalities, especially mass spectrometry (MS), have long dominated the quantitative analysis of illicit drug substances in various biological matrices [23,24]. In particular, gas chromatography hyphenated to mass spectrometry (GC−MS) is routinely utilised for this purpose and widely recognised as the gold standard for toxicology analysis [25,26], due to its ease of use, high specificity, and sensitivity [27,28]. However, the presence of numerous matrix-related interfering compounds often compromises chromatographic resolution, and the limited peak capacity in one-dimensional (1D) GC separation diminishes the quality of analytical findings [29,30,31].

The emergence of comprehensive two-dimensional GC (GC×GC) as a powerful bioanalytical separation tool shows great promise, offering significantly higher peak capacity through the ultilisation of two distinct separation mechanisms [32,33,34,35]. By expanding the separation space and increasing resolving power, GC×GC overcomes the limitations of GC−MS, thereby improving the separation and identification of psychoactive substances and their metabolites [36,37]. A major challenge in detecting NPS arises from the diverse chemical classes and the dynamic characteristics of these substances, which are often illicitly synthesised through minor structural modifications to avoid detection [6,38]. GC×GC exhibits the capabilities of separating a wide range of structurally related components [20], thereby circumventing many of the limitations inherent in 1D GC methods, offering improved separation and identification of NPS in complex matrices. Additionally, the requirement for extensive sample pre-treatment to remove interferences may be minimised, enabling rapid, high-throughput analysis.

Although GC×GC has demonstrated superior capabilities in resolving target psychoactive substances from complex matrix interferences, studies on its application for quantitatively analysing psychoactive substances in urine remain limited [16]. Kueh et al. first reported the use of GC×GC−FID for screening of selected drugs and metabolites relevant to urine [39], while Mitrevski et al. applied GC×GC for screening anabolic agents in doping control and profiling illicit drugs [28,29,40]. These studies have largely focused on qualitative aspects of drug analysis and have not thoroughly assessed the quantitative capabilities of GC×GC for the determination of psychoactive substances in urine. In this work, we describe a high-resolution GC×GC method for the rapid quantitative determination of 12 psychoactive substances from various drug classes, including NPS (amphetamine, methamphetamine, mephedrone, 3,4-methylenedioxyamphetamine (MDA), MDMA, PVP, norketamine, ketamine, ephylone, MDPV, methadone, and cocaine). The developed method was validated following the guidelines defined by Standard Practices for Method Validation in Forensic Toxicology, and its analytical practicality was demonstrated through the quantitative determination of these psychoactive substances in several spiked urine samples.

## 2. Materials and Methods

### 2.1. Chemicals and Reagents

Certified reference materials of amphetamine, methamphetamine, mephedrone, 3,4-methylenedioxyamphetamine (MDA), 3,4-methylenedioxymethamphetamine (MDMA), α-pyrrolidinovalerophenone (PVP), 3,4-methylenedioxypyrovalerone (MDPV), and methadone were purchased from Lipomed (Arlesheim, Switzerland). Norketamine was purchased from Cerilliant (Round Rock, TX, USA). Cocaine was purchased from Toronto Research Chemicals (Toronto, ON, Canada). *n*-Ethylpentylone (ephylone) was purchased from Chiron AS (Trondheim, Norway). Methaqualone (internal standard IS) and ketamine were provided by the Chemistry Department of Malaysia (Petaling Jaya, Malaysia). Dichloromethane (AR grade), sodium hydrogen carbonate (AR grade), sodium hydroxide (AR grade), and methanol (HPLC grade) were purchased from Merck (Darmstadt, Germany). A buffer solution (pH 12) was prepared by dissolving sodium hydrogen carbonate (8.5 g) and sodium hydroxide (4.6 g) in 1 L of distilled water.

### 2.2. Preparation of Standards

Primary stock solutions (100 or 1000 µg mL^−1^) of psychoactive substances were prepared using certified reference materials or authentic standards. The primary stock solutions were diluted in methanol to obtain 10 µg mL^−1^ of the mixed standards solution. An internal standard solution was prepared by diluting methaqualone in methanol to obtain a 10 µg mL^−1^ solution. All prepared stock solutions were kept in amber glass bottles and stored at 4 °C.

### 2.3. Sample Preparation

Blank human urine samples were obtained from volunteers in the laboratory. Approximately 1 mL of the collected drug-free urine samples were transferred into 15 mL glass tubes. Fortified urine samples were prepared by spiking the urine with an appropriate quantity of working standards solution to prepare matrix-matched samples at 9 different concentrations: 20, 50, 100, 200, 400, 600, 800, 1000, and 1500 ng mL^−1^. A 25 µL aliquot of the internal standard (methaqualone, 10 µg mL^−1^) solution was added into each tube to obtain a final concentration of 25 ng mL^−1^. A total of 500 µL of the buffer solution (pH 12) was added to the fortified urine samples. Subsequently, 150 µL of dichloromethane was added rapidly to the solution. The sample was then vortexed for 15 s and centrifugated at 3500 rpm for 8 min. The sedimented organic phase was then transferred into a glass vial containing an insert vial for injection into the GC−MS or GC×GC−FID. All the samples were analysed within 48 h from the completion of the extraction.

### 2.4. Instrumentation

#### 2.4.1. GC−MS System

GC−MS analyses were conducted on an Agilent Technologies 7890B GC system (Agilent Technologies, Santa Clara, CA, USA) equipped with a 5977B GC−MS single quadrupole mass spectrometer, 7693A auto sampler, and split/splitless inlet. The separation was carried out using a DB-5ms (95% methyl phenyl arylene polymer) capillary column of dimensions 30 m × 0.25 mm I.D. × 0.25 µm film thickness (Agilent Technologies). The initial oven temperature was 45 °C, then increased to 250 °C at 40 °C min^−1^ (hold 1 min) and increased to final temperature of 320 °C (hold 2 min) at 12 °C min^−1^. The injector temperature was 250 °C, and high-purity helium (99.999%; Alpha Gas Solution Sdn. Bhd., Malaysia) with a constant flow rate of 1 mL min^−1^ was used as the carrier gas. GC injection volume was 2 µL using pulsed splitless mode at 20 psi (hold 1 min); MS transfer line temperature was 280 °C; ion source temperature was 230 °C; quadrupole temperature was 150 °C, and the mass scan range of 40–550 Da was used.

#### 2.4.2. GC×GC−Flame Ionisation Detector (FID) System

GC×GC analyses were conducted on an Agilent Technologies 7890B GC system (Agilent Technologies) equipped with a flame ionisation detector (FID), an auto sampler, and a split/splitless inlet. The chromatographic separation was performed using a BP5MS column (30 m × 0.25 mm × 0.25 µm d_f_; Trajan Scientific and Medical, Ringwood, Australia) as the first-dimension column (^1^D) and a Supelcowax^®^10 column (2.5 m × 0.1 mm × 0.1 µm d_f_; Supelco, Bellefonte, PA, USA) as the second-dimension column (^2^D), connected by a deactivated pressfit (Restek Corporation, Bellefonte, PA, USA). The oven was initially held at 45 °C for 1 min, then increased to 275 °C at 45 °C min^−1^ and held for 3.5 min. The injector and detector temperatures were 180 °C and 280 °C, respectively, and the FID sampling frequency was 200 Hz. Helium was used as the carrier gas with a constant flow rate of 1.1 mL min^−1^. The sample introduction conditions were as follows: an injection volume of 2 µL in splitless mode with a splitless time of 2 min. A solid-state modulator (SSM) based on thermoelectric cooling (J&X Technologies, Shanghai, China) was used. The SSM entry zone temperature program emulated the GC oven settings. The SSM trap zone was initially reduced from 9 °C to −10 °C at a rate of 50 °C min^−1^ and held for 2.95 min, then heated back to 9 °C at 20 °C min^−1^ and held for 5.33 min. The SSM exit zone was initially held at 75 °C for 1 min, then ramped to 305 °C at 45 °C min^−1^ and held for 3.5 min. A modulation period (*P*_M_) of 5 s was used, although other *P*_M_ settings were also evaluated.

### 2.5. Validation of Analytical Method

The GC×GC−FID method was validated according to the Standard Practices for Method Validation in Forensic Toxicology guidelines [41]. The linearity of the calibration plots was studied using standard mixtures of the 12 drugs listed in Section 2.1, fortified in blank urine at nine concentration levels (20, 50, 100, 200, 400, 600, 800, 1000, and 1500 ng mL^−1^). The limit of detection (LOD) was determined with triplicate analysis of fortified blank urine samples at decreasing concentrations and estimated at concentrations with analyte peak intensity greater than 3.3 times the noise. The lower limit of quantitation (LLOQ) was estimated at the lowest non-zero calibrator at which the compound can be quantified confidently (within the acceptable bias% and CV%, <20%). Within-run precision (repeatability) and between-run precision (reproducibility) were determined using triplicate analyses of 100, 450, and 900 ng mL^−1^ concentrations over five different analyses and calculated using the one-way analysis of variance (ANOVA) approach using the following expressions, with mean square within groups (MS_wg_) and mean square between groups (MS_bg_) [41]:

(1) $$Within-run\;CV\;\left(\%\right)=\left[\frac{\sqrt{{MS}_{wg}}}{grand\;mean\;for\;each\;concentration}\right]\times \;100$$

(2) $$Between-run\;CV\;\left(\%\right)=\left[\frac{\sqrt{\frac{{MS}_{bg}+\left(n-1\right){MS}_{wg}}{n}}}{grand\;mean\;for\;each\;concentration}\right]\times \;100$$

Recovery calculated as the percentage (%) of the analyte response relative to the nominal values was determined by spiking blank urine at 100, 450, and 900 ng mL^−1^ concentration levels [42]. Bias was calculated at the same concentration levels using the following expression [41]:

(3) $$Bias\;\left(\%\right)\;at\;{Concentration}_{x}=\left[\frac{{Grand\;Mean\;of\;Calculated\;Concentration}_{x}-{Nominal\;Concentration}_{x}}{{Nominal\;Concentration}_{x}}\right]\times 100$$

### 2.6. Data Handling

For GC−MS analysis, data acquisition and processing were performed using the Agilent Masshunter version B07.00 software (Agilent Technologies). Compound identification and spectrum library matching were performed using the National Institute of Standards and Technology’s (NIST; Gaithersburg, MD, USA) 20 mass spectral library. For GC×GC−FID analysis, data acquisition and processing were performed using the Agilent Masshunter version 10.0 software (Agilent Technologies). Solute identification in GC×GC was performed based on the injection of respective standards. Contour plots were generated and processed using Canvas Panel version W1.8.0.29165 (J&X Technologies). Statistical analyses were performed using Microsoft Excel Version 2408 (Microsoft Corporation, Redmond, WA, USA).

## 3. Results and Discussion

### 3.1. GC−MS Analysis of Psychoactive Drugs

**Figure 1:**
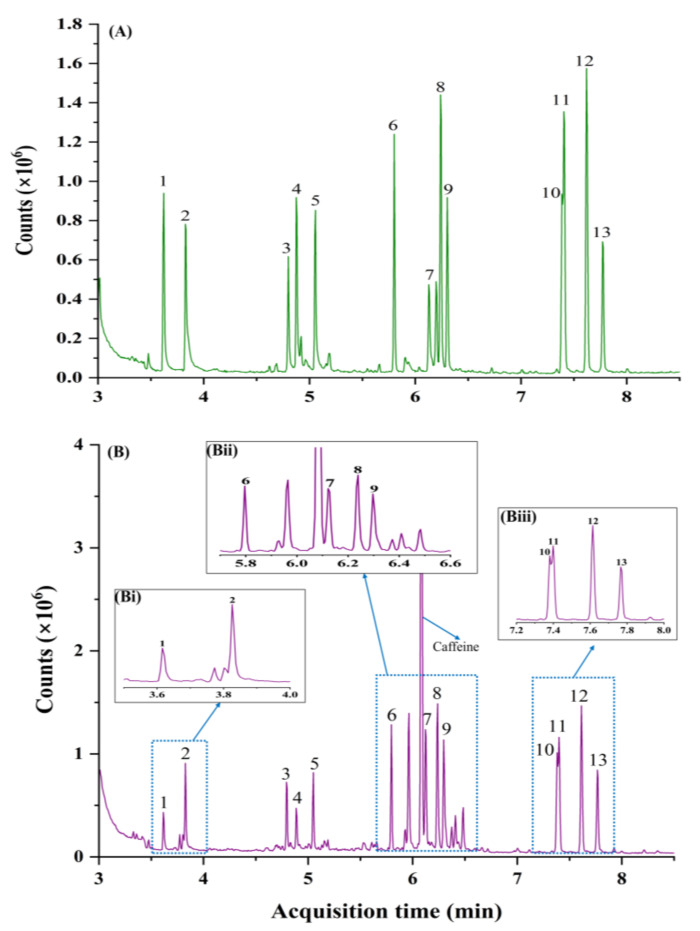
Total ion chromatograms of: (**A**) pure dilute standards solution, (**B**) fortified urine sample with spiked concentrations of 200 ng mL^−1^, respectively, and (**Bi**) expansion of selected chromatographic region from 3.5 to 4.0 min in (**B**), (**Bii**) expansion of selected chromatographic region from 5.7 to 6.6 min in (**B**), and (**Biii**) expansion of selected chromatographic region from 7.2 to 8.0 min in (**B**). Peaks: 1, amphetamine; 2, methamphetamine; 3, mephedrone; 4, MDA; 5, MDMA; 6, PVP; 7, norketamine; 8, ketamine; 9, ephylone; 10, MDPV; 11, methadone; 12, methaqualone (IS); 13, cocaine.

GC−MS is routinely used in toxicology for the determination of xenobiotics in biological specimens through the screening and mass spectrum matching of compounds against databases such as NIST. A major challenge in determining psychoactive substances in biological samples is the presence of numerous interfering compounds that potentially co-elute with analytes of interest, impeding trace-level detection. Initially, a urine sample fortified with psychoactive substances (200 ng mL^−1^) was analysed using GC−MS (full scan mode). Figure 1 illustrates the total ion chromatograms of 12 psychoactive substances, including amphetamine-type stimulants, opioids, NPS, and others, in a pure dilute standard solution and also standards fortified in blank urine samples. The non-polar phenyl arylene stationary phase provided somewhat satisfactory separation, with most of the analytes showing adequate resolution. However, significant co-elutions (Figure 1Bii) were observed for norketamine (peak 7) and caffeine, and MDPV (peak 10) and methadone (peak 11) spiked in blank urine (Figure 1Biii), with resolution (R_s_) values of 0.9 and 0.5, respectively. Notably, MDPV and methadone have unique ion masses at *m*/*z* 126 and *m*/*z* 72, respectively, suggesting that these compounds can be resolved through an extracted ion chromatogram or selective ion monitoring mode. The presence of caffeine in the urine sample might be attributed to the consumption of food products such as coffee, tea, chocolate, etc. with its metabolites (theophylline and theobromine) frequently found in biological specimens [43,44]. Results (Figure 1Bi) also indicated partial co-elution between methamphetamine and an unknown peak, R_s_ value of 0.5. The lack of separation of these targeted compounds from matrix interferences can potentially result in incorrect determination of their concentration levels. Additionally, overlapping peaks often result in poor mass spectrum database matches, potentially obscuring trace-level compound identification. Nevertheless, the observed co-elutions are a major limitation of 1D GC due to its limited peak capacity and insufficient phase selectivity in single column analyses. Thus, accurate quantification of these compounds would necessitate additional chromatographic or MS deconvolution processes. Isolating compounds of interest from interferences, followed by separation and identification, is crucial for analysing biological samples, given their complexity and the diversity of compounds present [30,45].

### 3.2. Evaluation of GC×GC Conditions for Separation of Psychoactive Compounds

**Figure 2:**
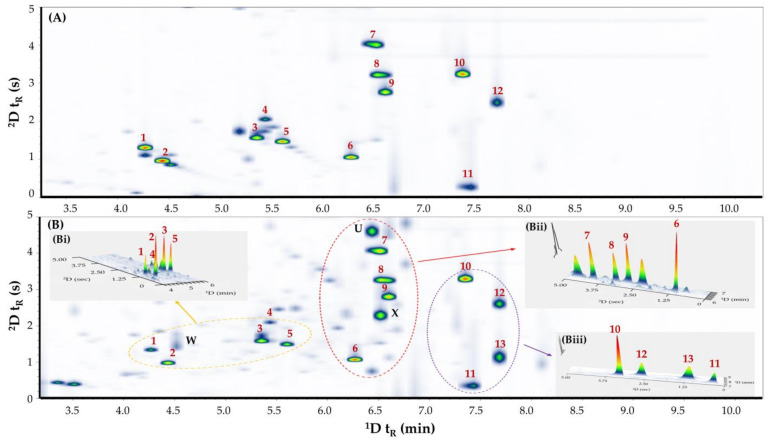
GC×GC−FID contour plots of (**A**) mixed standards in (diluted) neat solution fortified with psychoactive substances (200 ng mL^−1^), (**B**) representative urine sample fortified with psychoactive substances (400 ng mL^−1^), (**Bi**) Extracted 3D plots of selected ^1^D time interval 4–6 min in (**B**), (**Bii**) Extracted 3D plots of selected fractions in ^1^D (6–7 min) in (**B**), and (**Biii**) Extracted 3D plots of selected ^1^D time interval 7–9 min in (**B**). Peaks: 1, amphetamine; 2, methamphetamine; 3, mephedrone; 4, MDA; 5, MDMA; 6, PVP; 7, norketamine; 8, ketamine; 9, ephylone; 10, methadone; 11, MDPV; 12, cocaine; 13, methaqualone; X, caffeine; U, unknown; W, unknown.

Given the improved peak capacity of GC×GC, this high-resolution separation technique was evaluated for the separation of ATS, opioids, NPS, and others in urine samples. Initially, two column sets were assessed in this study, comprising non-polar/polar (BP5MS × Supelcowax^®^10) and polar/non-polar (MEGA-Wax HT × SLB-5ms) configurations. The separation performance of these column combinations is depicted in Figure S1 (Supplementary Information). The combination of the ^1^D non-polar column and the ^2^D polar column provided a better spread of the psychoactive solutes across the 2D space. Importantly, this column arrangement showed improved separation with no co-elution observed for all psychoactive solutes when compared to the separation achieved using the polar/non-polar arrangement. Thus, this set was selected for further study. Next, the effect of modulation period (*P*_M_; 2–6 s) was investigated. Results indicated that *P*_M_ < 5 s causes significant wraparound that affects the solute separation, particularly in urine samples. On the contrary, *P*_M_ > 5 s causes undersampling of ^1^D peaks, which reduces the resolution of ^1^D separation. Thus, a *P*_M_ of 5 s was chosen for further analysis. Using the selected non-polar/polar combination and a 5 s *P*_M_, all analytes were baselined and separated within 8 min, with average peak widths at a baseline of 350 ms. Figure 2 illustrates the separation of psychoactive solutes in both the standard solution and the fortified urine sample. By using a polar ^2^D wax column (Figure 2A, baseline separation (*R*_s_ ≥ 1.5) was achieved for norketamine (peak 7), ketamine (peak 8), ephylone (peak 9), methadone (peak 10), and MDPV (peak 11). Notably, norketamine, ketamine, and ephylone were effectively separated from caffeine (peak X) and an unknown interfering compound (peak U) that otherwise co-eluted on the ^1^D non-polar column. Additionally, an unknown interfering compound (peak W), partially co-eluting with methamphetamine in some urine samples, was also resolved. This highlights the unprecedented resolving power of GC×GC in untangling analytes of interest in complex biological matrices without the need for extensive sample preparation procedures.

### 3.3. Validation Studies

#### 3.3.1. Linearity

The linearity of the calibration curves was studied using matrix-matched standard mixtures at nine concentration levels (20, 50, 100, 200, 400, 600, 800, 1000, and 1500 ng mL^−1^) with methaqualone (25 ng mL^−1^) as IS. As shown in Table 1, good linearity was observed for all compounds. Correlation coefficients r^2^ above 0.99 were obtained, indicating excellent linearity over the concentration range studied. The method was determined to be linear for all analytes over the range from LLOQ to 1500 ng mL^−1^.

#### 3.3.2. Limits of Detection and Lower Limits of Quantitation

The limits of detection (LOD) and lower limits of quantitation (LLOQ) for the psychoactive substances were determined as the lowest concentrations producing identifiable peaks, with signal responses at least 3.3 times greater than the background noise. The estimated LODs and LLOQs for the spiked psychoactive substances in blank urine samples are detailed in Table 1, demonstrating good sensitivity with LODs ranging from 10 to 20 ng mL^−1^ and LLOQs determined at 50 ng mL^−1^. The GC×GC−FID method exhibited comparable LODs and LLOQs to reported GC−MS methods that use different sample preparation techniques (Table 2), including solid phase extraction (SPE) with pentafluoropropionic anhydride derivatisation [46,47], dispersive liquid-liquid microextraction (DLLME) with hexyl chloroformate derivatisation [48], and liquid-liquid extraction (LLE) with trifluoroacetic anhydride derivatisation [49]. Although these methods have achieved low detection limits, they require more stringent sample preparation workflows and longer analysis time (>30 min). To circumvent the longer GC−MS analysis times of derivatisation followed by extraction approaches, it is possible to use simultaneous scans and selected ion modes to lower the detection limits by 10-fold to 50-fold for most of the drug analytes. The low LODs obtained using the GC×GC−FID method can be attributed to the focusing effect of the modulation process. The effective solute trapping and remobilisation between the cooling and heating stages of the modulator resulted in sharper chromatographic peaks and improved sensitivity [50,51]. In this study, underivatized analytes were studied. In our experience, this is suitable when new column phases are used. Derivatisation may be necessary when older columns are used, at the expense of a longer method work-up.

#### 3.3.3. Precision, Bias and Recovery

Precision and bias evaluated at three concentration levels (100, 450, and 900 ng mL^−1^) were within the acceptance threshold of <15% [41], indicating satisfactory repeatability, reproducibility, and systematic error (determined as bias) of the method. Table 3 summarises the precision, bias, and recoveries of the psychoactive compounds analysed using GC×GC−FID. Within-run precision (CV%) for peak areas ranged from 1.5% to 11.4%, and between-run precision ranged from 1.2% to 10.3%. Bias (maximum acceptable threshold ±20%, [41]) ranged from −3.1% to 14.5%. Retention time precision (CV%) for the ^1^D ranged from 0.001% to 0.3% within-run and 0.02% to 0.75% between-run, while for the ^2^D, it was 0.3–1.9% within-run and 0.8–6.1% between-run. Additionally, good recovery percentages were achieved with values ranging from 96.9% to 114.5%. The GC×GC−FID method showed improved precision and recoveries compared to GC−MS methods from other studies, which reported ranges of 0.4–14.9% (CV%) and 92–115% [48], 2.8–18.6% (CV%) and 64–105% [47], and 0.8–12.5% (CV%) and 69–96% [54].

### 3.4. Analysis of Spiked Urine Samples

**Figure 3:**
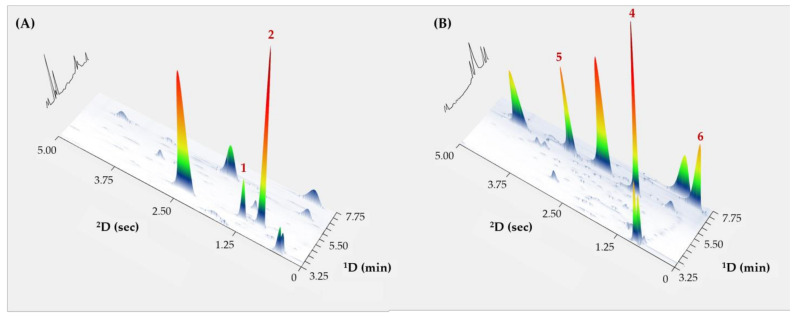
GC×GC−FID extracted 3D plots of selected fortified urine samples. (**A**) fortified sample number 1; and (**B**) fortified sample number 6. Peaks: 1, amphetamine; 2, methamphetamine; 4, PVP; 5, ephylone; 6, MDPV.

The validated method was applied for the determination of psychoactive substances in several spiked urine samples with varying compositions and concentration levels. Figure 3 presents extracted 3D chromatograms of illicit drugs spiked into a urine background matrix. The target drug compounds were well-resolved from the matrix components and readily identified in the 2D separation space based on their respective ^1^D and ^2^D retention times. In this instance, it can be appreciated that GC×GC contour plots can serve as a qualitative screening tool, where the appearance of contours (i.e., peaks) within defined 2D retention windows would indicate the presence of drug metabolites, akin to drug fingerprinting. Furthermore, the determined concentrations were found to be closely correlated with the spiked levels (Table 4), exhibiting recovery percentages ranging from 91.9% to 107.4%. These findings successfully highlight the qualitative and quantitative prospects of the GC×GC−FID approach for analysing illicit substances, which will be useful for forensic toxicologists.

## 4. Conclusions

This study demonstrated the applicability of a fast GC×GC−FID approach for the rapid determination of 12 psychoactive substances in urine samples without necessitating complicated or time-consuming sample preparation protocols. The comprehensive nature of GC×GC enabled simultaneous analysis of multiple illicit psychoactive compounds from different classes in urine samples in less than 8 min. The chromatographic features of two independent separation mechanisms to delineate targeted analytes from the background urine matrix are illustrated. The developed method showed good validation data (linearity, detection limits, precision, bias, and recoveries) for the accurate measurement of these drugs in urine matrix, with the performance criteria satisfying the Standard Practices for Method Validation in Forensic Toxicology. The proposed method demonstrates notable advantages over classical 1D GC, offering better separation selectivity and signal enhancement, making it a promising alternative for illicit drug testing. A further benefit, especially when GC×GC−MS with TIC data collection, is the prospect of archiving data for future reference for identifying additional drugs of abuse, should these be of interest at a later date.

## Figures and Tables

**Table 1:** Linearity ranges, limits of detection, and lower limits of quantification of the studied psychoactive substances using GC×GC−FID.

Compound	Class of Drug	r^2^	Linearity Range (ng mL^−1^)	LOD (ng mL^−1^)	LLOQ (ng mL^−1^)
Amphetamine	Amphetamine type stimulants	0.9931	50–1500	10	50
Methamphetamine	Amphetamine type stimulants	0.9960	50–1500	10	50
Mephedrone	New psychoactive substance	0.9934	50–1500	15	50
MDA	Amphetamine type stimulants	0.9915	50–1500	20	50
MDMA	Amphetamine type stimulants	0.9965	50–1500	15	50
PVP	New psychoactive substance	0.9974	50–1500	15	50
Norketamine	Dissociative anesthetic	0.9968	50–1500	20	50
Ephylone	New psychoactive substance	0.9965	50–1500	20	50
Ketamine	Dissociative anesthetic	0.9976	50–1500	15	50
MDPV	New psychoactive substance	0.9977	50–1500	20	50
Methadone	Opioid	0.9973	50–1500	10	50
Cocaine	Stimulant	0.9975	50–1500	15	50

Amphetamine, MDA, and norketamine are metabolites of methamphetamine, MDMA, and ketamine, respectively.

**Table 2:** Comparison of the current study with previous research on the analysis of illicit substances in urine.

Drug Classes	Number of Substances	Extraction Method	Analyte Detection Technique	LOD (ng mL^−1^)	LLOQ (ng mL^−1^)	Recovery (%)	Runtime (min)	Ref.
ATS, NPS. ketamine, methadone, cocaine	12	LLE	GC×GC−FID	10–20	50	96.9–114.5	9.6	Present work
Cathinones, ATS	29	SPE	GC−MS	0.5–10	5–50	80–120	25	[46]
NPS	23	SPE	GC-MS	0.2–1	0.5–20	64–105	30	[47]
Cathinones, ATS, NPS	26	DLLME	GC−MS	1–10	2–50	92–115	16.1	[48]
Cathinones	19	LLE	GC−MS	10–30	30–100	-	9.9	[49]
ATS. NPS, opioids, benzodiazepines, ketamine	37	Hydrolysis and LLE	LC-MS/MS	1–30	50	13.6–112.8	6	[52]
ATS. NPS, opioids, benzodiazepines, cocaine, ketamine	68	Dilution	LC-MS/MS	0.05–0.5	0.1–0.5	-	15	[53]

**Table 3:** Precision, bias, and recoveries of the psychoactive substances analysed using GC×GC−FID.

Compound	^a^ Within-Run Precision, CV (%)			^a^ Between-Run Precision, CV (%)			Bias (%)			Recovery (%)		
	100 ng mL^−1^	450 ng mL^−1^	900 ng mL^−1^	100 ng mL^−1^	450 ng mL^−1^	900 ng mL^−1^	100 ng mL^−1^	450 ng mL^−1^	900 ng mL^−1^	100 ng mL^−1^	450 ng mL^−1^	900 ng mL^−1^
Amphetamine	10.9	7.3	4.1	8.0	7.2	6.9	2.7	−2.8	3.8	102.7	97.2	103.8
Methamphetamine	8.3	6.4	5.6	7.0	6.5	6.0	4.6	2.5	3.6	104.6	102.5	103.6
Mephedrone	5.1	5.8	2.9	6.1	5.3	2.5	13.8	−1.1	2.1	113.8	98.9	102.1
MDA	9.4	5.7	3.6	10.3	6.7	4.3	1.5	−0.6	4.2	101.5	99.4	104.2
MDMA	11.4	5.3	3.0	8.3	6.1	3.8	0.6	6.0	6.0	100.6	106.0	106.0
PVP	6.8	2.7	2.1	5.5	3.1	2.5	7.1	−3.1	1.5	107.1	96.9	101.5
Norketamine	5.7	3.0	3.1	9.2	2.6	3.1	5.5	3.5	4.5	105.5	103.5	104.5
Ephylone	10.3	4.7	2.3	7.8	4.0	4.7	9.3	−0.7	1.5	109.3	99.3	101.5
Ketamine	6.5	3.5	2.4	6.2	2.8	2.5	7.7	2.0	3.4	107.7	102.0	103.4
MDPV	8.0	2.0	2.1	5.2	2.8	2.8	14.5	0.4	5.0	114.5	100.4	105.0
Methadone	7.1	2.9	2.0	6.6	1.9	1.2	12.3	1.5	1.3	112.3	101.5	101.3
Cocaine	4.7	1.8	1.5	3.5	3.0	4.7	8.8	−1.6	−0.9	108.8	98.4	99.1

^a^ Within-run and between-run precision, CV(%) was assessed using peak areas.

**Table 4:** Recovery percentages of fortified urine samples using the proposed GC×GC−FID method.

Sample	Compounds	Spiked Concentration (ng mL^−1^)	Mean Calculated Concentration (ng mL^−1^)	Recovery (%)
1	Amphetamine Methamphetamine	300.0 700.0	282.5 ± 7.8 658.5 ± 12.0	94.2 ± 2.6 94.1 ± 1.7
2	Ketamine Norketamine	600.0 300.0	593.0 ± 8.5 317.0 ± 7.1	98.8 ± 2.4 105.8 ± 1.4
3	MDMA MDA	700.0 150.0	643.5 ± 34.6 140.5 ± 4.9	91.9 ± 4.9 93.4 ± 3.3
4	Methadone	500.0	533.0 ± 0.1	106.5 ± 0.1
5	Amphetamine Methamphetamine	250.0 600.0	258.0 ± 5.7 557.0 ± 7.1	103.2 ± 2.3 92.8 ± 1.2
6	PVP Ephylone MDPV	350.0 350.0 400.0	344.5 ± 2.1 331.0 ± 4.2 382.5 ± 12.0	98.4 ± 0.6 94.5 ± 1.2 95.7 ± 3.0
7	Mephedrone Cocaine	330.0 400.0	330.5 ± 37.5 429.5 ± 6.4	100.1 ± 11.4 107.4 ± 1.6

## Data Availability

The raw data supporting the findings of this study will be made available by the authors on request.

## Supplementary Materials

The following supporting information can be downloaded at: https://www.mdpi.com/article/10.3390/metabo14110643/s1, Figure S1: GC×GC−FID contour plots of 300 ng mL^−1^ mixed standards fortified urine samples obtained using two-column configuration sets (A) BP5MS ^1^D column and Supelcowax^®^10 ^2^D column, and (B) MEGA-Wax HT ^1^D column and SLB-5ms ^2^D column.
