# Supplementary material for: Rapid Determination of Methamphetamine, Methylenedioxymethamphetamine, Methadone, Ketamine, Cocaine, and New Psychoactive Substances in Urine Samples Using Comprehensive Two-Dimensional Gas Chromatography
Source: Metabolites. 2024 Nov 20;14(11):643. doi: 10.3390/metabo14110643 (PMC11596927; doi:10.3390/metabo14110643)
Supplement: Supplementary file 1 [file metabolites-14-00643-s001.zip › metabolites-3290980-supplementary.pdf]

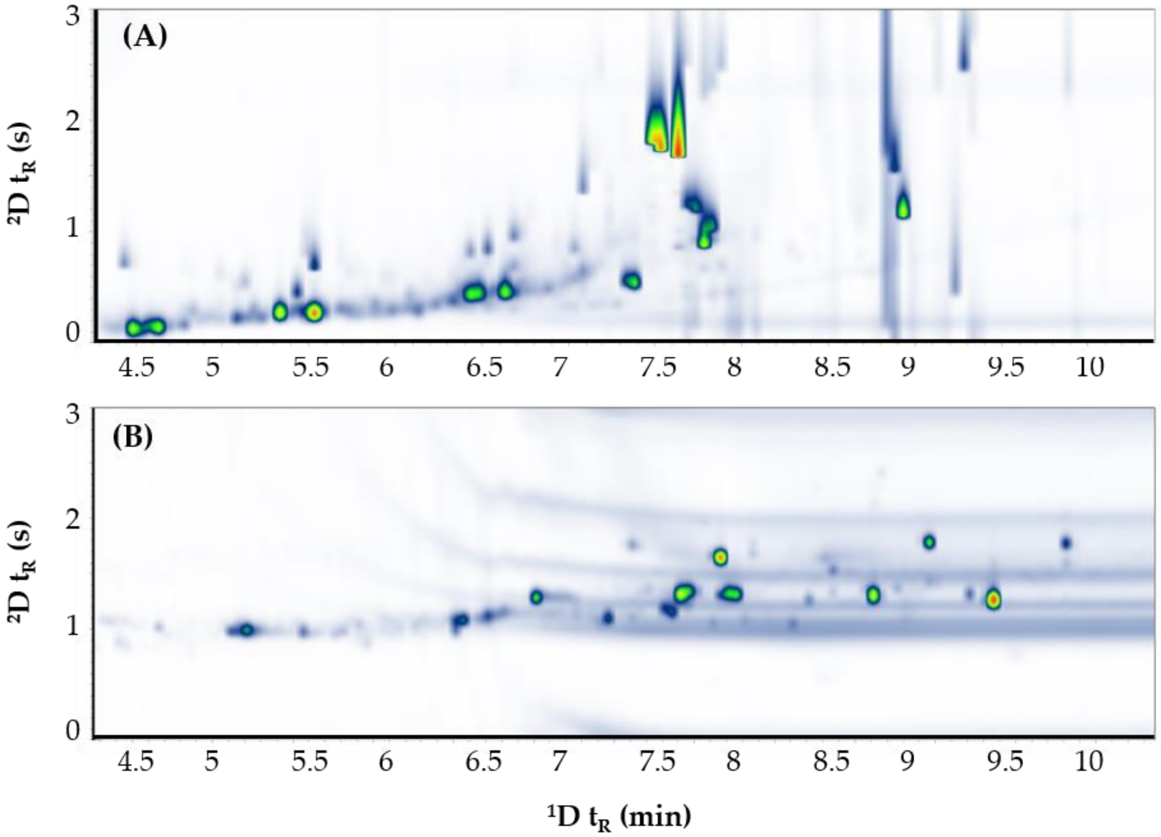

Figure S1 GC×GC–FID contour plots of 300 ng mL^{-1} mixed standards fortified urine samples obtained using two column configuration sets (A) BP5MS ^{1}D column and Supelcowax®10 ^{2}D column, and (B) MEGA-Wax HT ^{1}D column and SLB-5ms ^{2}D column.
